# Comparative Evaluation of Marginal Leakage of Various Bevel Designs Using Direct Composite Restoration in Fractured Anterior Teeth: An In Vitro Study

**DOI:** 10.7759/cureus.56860

**Published:** 2024-03-25

**Authors:** Shruthi Rajagopal, Subash Sharma

**Affiliations:** 1 Department of Conservative Dentistry and Endodontics, Saveetha Dental College and Hospitals, Saveetha Institute of Medical and Technical Sciences (SIMATS) Saveetha University, Chennai, IND

**Keywords:** polymerization shrinkage, direct composite restoration, technology, health innovation, quality of life, ellis class ii fractures, bevel, composite

## Abstract

Background and objective

Resin-based restorative materials are the backbone of modern restorative dentistry. But in spite of being an excellent material, there are many shortcomings with direct resin restorative materials such as polymerization shrinkage and microleakage that complicate the rate of clinical success. Hence, the aim of the present study is to compare the microleakage caused by composite restorations using two different bevels, primary and zigzag bevels, while restoring fractured anterior teeth.

Materials and methodology

Thirty non-carious maxillary central incisors were split into two divisions wherein Group I received the primary bevel and Group II received the zigzag bevel. After receiving the bevel, the samples are restored with direct filling composite material (Neo Spectra ST, Dentsply Sirona, Charlotte, NC, USA). The restored samples underwent thermocycling (Holmarc, Kochi, India) and were assessed for microleakage under a stereomicroscope (Leica M205, Wetzlar, Germany). For the statistical analysis, IBM SPSS Statistics for Windows, V. 23.0 (IBM Corp., Armonk, NY) was used. Descriptive statistics were expressed in mean and standard deviation. Analytical statistics including the independent Student t-test was used to assess the difference derived from both groups at p<0.05. The normality of the data was assessed using the Shapiro-Wilk test.

Results

In the primary bevel, 53.3% of the samples showed first-degree microleakage, and 46.7% showed second-degree microleakage, respectively, and in samples restored using the zigzag bevel, 66.7% of the samples had no microleakage, and 33.3% of the samples had first-degree microleakage. The independent t-test revealed that the microleakage of the zigzag bevel showed a significant difference, being superior to the primary bevel at p<0.01.

Conclusion

Acknowledging the limitations of the study conducted, both bevel designs had a certain degree of microleakage when restored with composite material in anterior fractured teeth. However, the zigzag bevel produced significantly lesser microleakage as compared to the primary bevel restorations.

## Introduction

Since the advent of restorative and adhesive dentistry, discovering materials that are the aesthetic and functional replacement of any cavity surface following dental caries excavation, thereby replacing natural tooth structure has been the major focus of dentists across the globe. The choice of material would depend after evaluating clinical factors like the type and extent of the cavity, along with patient compliance, material properties, and evidence-based protocols [1].

The three key restorative materials for direct restorations utilised historically have been amalgam, glass ionomer, and composite [2]. The American dentist, Greene Black, was the first to formulate the concepts of cavity preparation for restorations with amalgam material. The fundamental ideas from Black's epoch still apply to conservative tooth preparation for certain clinical scenarios. The most notable protocols dictate various principles for resistance and retention forms, the most notable one being the cavosurface margin at a butt joint [3].

Alternatively, with the advent of composite resins, these conventional cavity designs have been made redundant. Composite resin materials can be used to replace cavities of any form or extent, thereby being more conservative. Additionally, there is no necessity for the cavosurface margin to be placed at the butt joint.

Hence, there has been a rampant increase in studies conducted in recent years, regarding the application of tooth-coloured resin materials due to the need for superior aesthetics along with the conservation of the remaining tooth structure. In contemporary dentistry, composite materials are the most utilised due to their superior mechanical and aesthetic properties. These materials have improved physical properties in terms of compressive strength, aesthetics, and predictability. The present composite formulas have been enhanced, showing superior wear resistance along with the preservation of tooth structure. They have the potential for tooth reinforcement and a reduced need for the use of additional dental materials when used in conjunction with the appropriate adhesive systems.

Amongst the many disadvantages, polymerization shrinkage is the most common phenomenon that takes place during the curing process of these resin materials. It is the contraction of the polymer matrix as it changes through various physical states. This can lead to several complications in the performance and durability of composite materials [4].

One major disadvantage of polymerization shrinkage is the potential for cracking and deformation. As the polymer matrix shrinks, it can put stress on the reinforcement fibres of the material and cause cracks. This can lead to a loss of strength and stiffness in the composite material. Additionally, shrinkage can cause the composite to warp or bend, which can negatively impact its ability to function in the oral cavity.

Another disadvantage of polymerization shrinkage is the potential for the development of voids or porosity in the finished composite restoration. As the polymer matrix shrinks, it can pull away from the reinforcement fibres and create spaces or voids in the material. This weakens the composite and makes it more susceptible to damage. Additionally, polymerization shrinkage can make it difficult to achieve precise dimensions in the restoration. As the composite shrinks during curing, it can be challenging to maintain and control the final shape of the material, thereby reducing the clinical longevity of such restorations [5].

As a factor of polymerization shrinkage, the main deleterious effect seen is microleakage. The formation of microcracks, due to the use of a non-incremental layering technique or the use of insufficient finishing and polishing techniques, is a factor that can cause microleakage, which is characterised by minute gaps present at the interface region between the tooth and the restorative material which leads to the degradation of the bond strength of the material over a period of time [6].

One way to combat this, in order to enhance retention and aesthetics, is the use of bevel in tooth preparations, especially for anterior composite restorations due to the high demand for aesthetics in this region. In order to decrease stresses at the bonding interface, increase the surface area for bonding to the enamel, and improve the shade matching by camouflaging the demarcation between the tooth surface and the restoration, a bevel is given after the carious removal. It is a procedure that entails generating a little chamfer or feather at the restoration's edge [7]. Bevel designs involve contours prepared at the cavosurface edge of the restoration that influences the aesthetics and durability of the restoration.

One common bevel design is the "primary bevel", which involves creating a 45-degree angle at the margin, permitting simple application of the composite material and minimal removal of healthy tooth structure. Another design is the shoulder bevel, which creates a butt angle at the cavosurface margin, providing a stronger bond for the composite restoration but at the cost of healthy tooth structure.

Another important aspect of the bevel design is the "depth" of the bevel, which refers to how much tooth structure is removed to create the bevel. A bevel of greater depth allows for better adaptation of the resin material but also requires more removal of sound tooth structure. In addition, the use of bevels in the cervical (neck) area of the tooth can help to prevent post-operative sensitivity by creating a physical barrier between the composite and the pulp chamber [8].

Thus, this study aims to find a correlation between the bevel design and degree of microleakage in order to ensure more clinical longevity and aesthetics of anterior direct composite restorations. The primary objective of this in vitro study is to evaluate and compare the effect of various bevel designs on marginal leakage for anterior fractured teeth restored with direct composite restoration.

## Materials and methods

The present study was carried out in the Department of Conservative Dentistry and Endodontics at Saveetha Dental College and Hospitals, Saveetha Institute of Medical and Technical Sciences (SIMATS), Saveetha University, Chennai, India, from September to October 2022.

The Institutional Human Ethical Committee of Saveetha Dental College approved the study (approval number: ECR/1698/Inst/TN/2022).

Inclusion criteria

The study involved the inclusion of human permanent non-carious maxillary central incisors that were freshly extracted. The extracted teeth involved periodontal problems. They were devoid of fracture lines or crazing, with intact enamel, dentin, and root structures present.

Exclusion criteria

The study involved the exclusion of human-extracted teeth with carious lesions, developmental disturbances, abnormal root formation, attrition, or teeth involving cervical abrasion. Sample size calculation was done using G*Power to determine the sample size based on the reference study [9], using a p-value of 0.05 and a 95% power with an effect size of 0.636. A sample size of 30 was included as a result of the sample size computation.

Specimen preparation 

Thirty teeth were obtained and underwent surface debridement with hand scalers to remove all adherent material and stains. The teeth were divided equally into two groups: Group I receiving the primary bevel and Group II receiving zigzag bevel.

For standardisation, all samples were mounted in the same fashion. The teeth were mounted using tubes made of polyvinyl chloride (PVC) tubing that were 3 cm long and 2.5 mm in diameter. In order to establish parallelism, the mounting was standardised with the cervical line of each sample, at the level of the poured acrylic resin during the mounting process. The edge of the tube and the tooth were at a 90° angle. Following that, the dough process was used to embed the mounted teeth's roots in cold-cure acrylic resin.

A mesio-incisal fracture line was subjected to all the samples, standardised measuring 3 mm distally along the incisal edge and 3 mm gingivally. They were then linked to create the base of a hypothetical triangle with the apex corresponding to mesio-incisal line angle.

Preparation of the bevel

For the preparation of the samples, intentional fractures were subjected to the samples using a low-speed diamond disc (ContacEZ, London, Canada) at a constant speed of 150 RPM. Using a standard diamond rotary bur (Prima Dental Predator turbo bur 850T-016C, Gloucester, UK), a primary bevel and zigzag bevel preparation were placed on the incisolabial aspect of the sample. 

**Figure 1:**
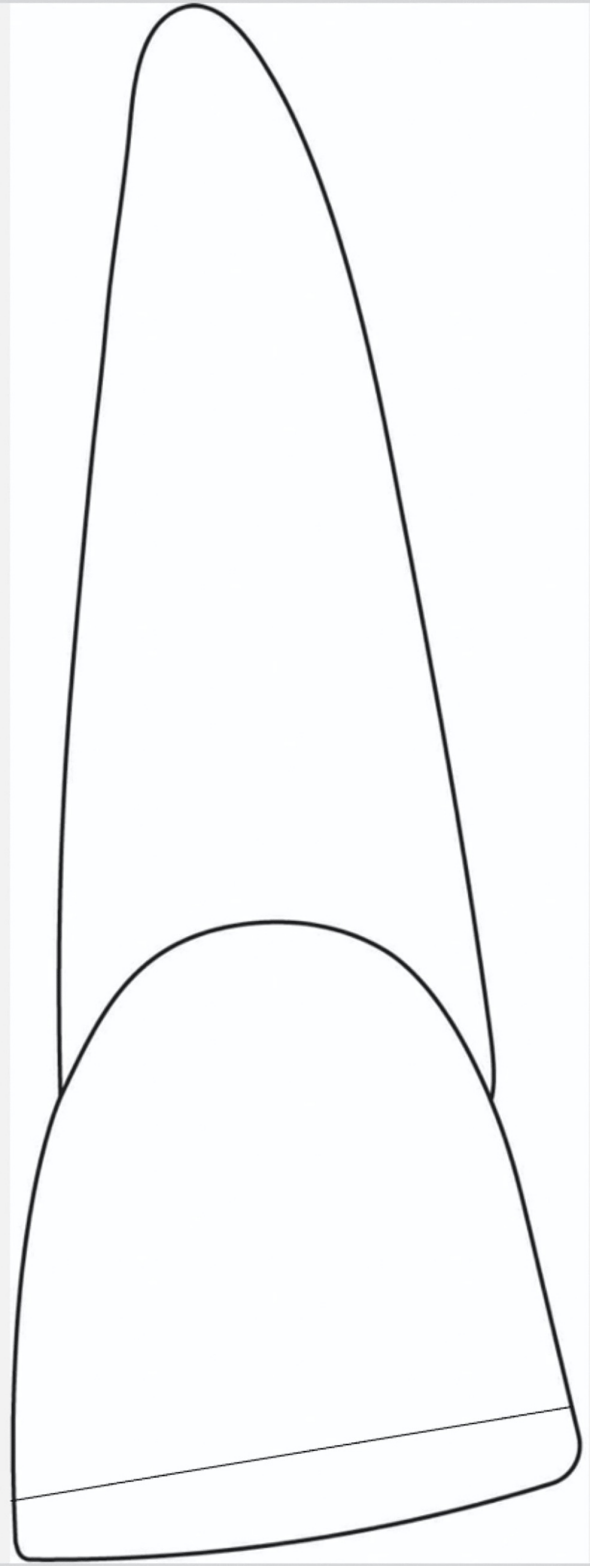
Schematic diagram of bevel design in Group I (primary bevel)

**Figure 2:**
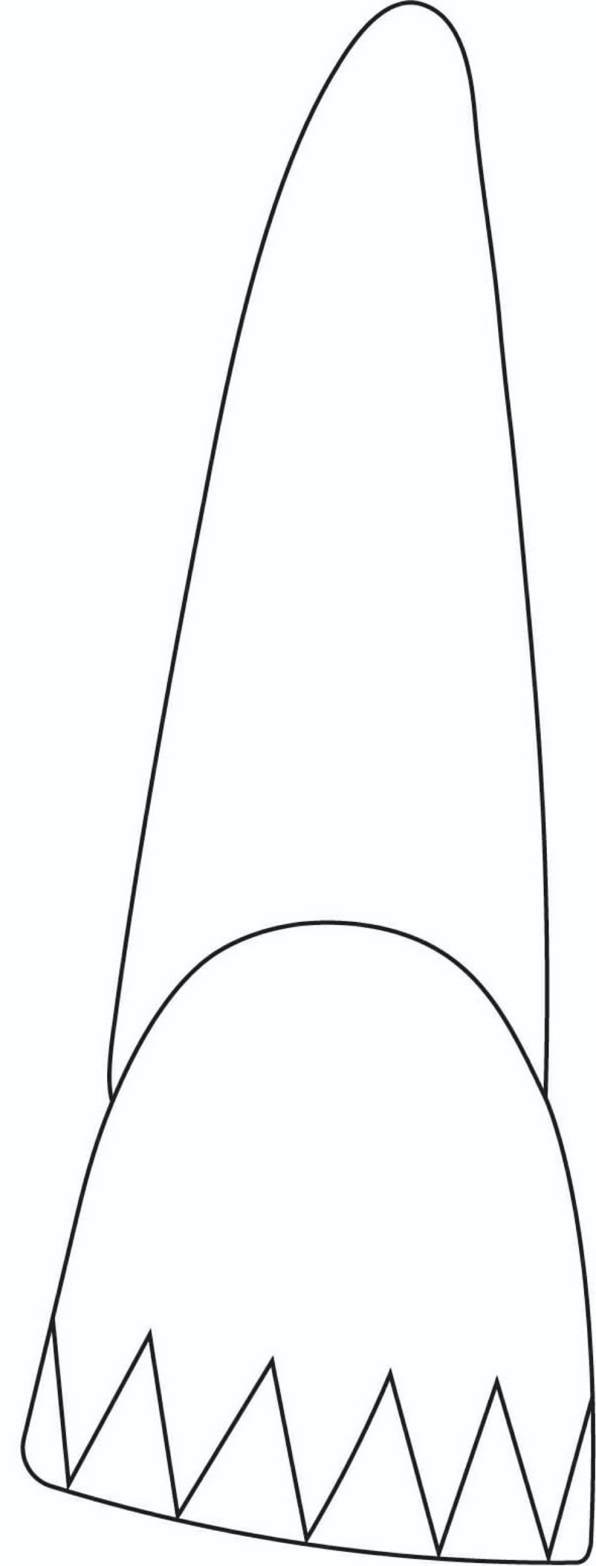
Schematic diagram of bevel design in Group II (zigzag bevel)

On the cavosurface margin of the tooth for the primary bevel group, a 45-degree bevel was provided that extended 2 mm past the fractured incisal edge and across the full thickness of enamel. For the second group, zigzag bevel preparation was placed extending 2 mm beyond the fractured incisal edge. The bevels are represented in Figure 1 and Figure 2, respectively.

Restoration of the fractured teeth with direct composite 

**Figure 3:**
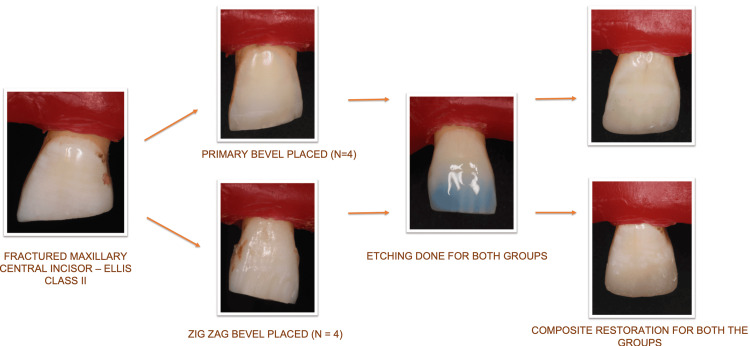
Workflow of the restorative procedure for both groups

The enamel margins for both groups were subjected to acid etching with 35% phosphoric acid gel (Ivoclar N Etchant, Schaan, Liechtenstein) for a time interval of 15 seconds, followed by a complete 15-second water rinsing. A single-component total-etch adhesive (Ivoclar Vivadent Te-econom Bond, Schaan, Liechtenstein) was applied using an adhesive applicator tip as directed by the manufacturer. It was then exposed to curing light for a time period of 20 seconds using an Ivoclar Bluephase PowerCure LED curing light (Ivoclar, Schaan, Liechtenstein) with an irradiance of 1,100 mW/cm^2^. Dentsply Neo Spectra ST (Dentsply Sirona, Charlotte, NC, USA) direct nanofilled composite was used to restore all of the preparations. The resin composites were cleaned of superfluous material before being polymerized using Bluephase light cure for a duration of 20 seconds [10]. The workflow for the same is depicted in Figure 3 [11].

Assessment of microleakage

Samples were kept immersed in distilled water. They were exposed to thermocycling (Holmarc, Kochi, India). This experiment employed temperatures of 12°C and 60°C. The restorations were subjected to 1500 cycles altogether. All tooth surfaces were covered with two coats of nail paint, with the exception of a 1 mm border surrounding the restoration. The teeth were exposed for four hours to a dye solution (Guerbet Ltd., Villepinte, France) containing 50% silver nitrate while being shielded from the light. The teeth were then submerged for four hours in a photographic film-developing solution (Spring Record Speed Fixer) while being illuminated by a 200-watt light source. The teeth were washed under tap water to rinse out the solution. The restoration was subsequently divided at its midway buccolingually to create specimens, with the exposure of the tooth interface from the cavosurface margin to the pulpal wall. The samples were examined under a stereomicroscope (Leica, Wetzlar, Germany) at a 10× magnification to determine the degree of marginal leakage using the following criteria provided by Khera and Chan [12] (Table 1).

**Table 1:** Criteria provided by Khera and Chan for appraising the degree of marginal leakage Reference: [12]

Criteria for appraising the degree of marginal leakage	
0°	No leakage
1°	Less than and up to one-half of the depth of the cavity preparation was penetrated by the dye
2°	More than one-half of the depth of the cavity preparation was penetrated by the dye but not up to the junction of the axial and occlusal or cervical wall
3°	Dye penetration was up to the junction of the axial and occlusal or cervical wall but did not include the axial wall
4°	Dye penetration included the axial wall

Statistical analysis

A spreadsheet on Microsoft Excel created was used to enter the raw data, and IBM SPSS Statistics for Windows, V. 23.0 (IBM Corp., Armonk, NY) was used to analyse it. Descriptive statistics were used to analyse the data, including frequency, percentages, mean, standard deviation, and 95% confidence range. The normality of the distribution of all the parameters was evaluated using the Shapiro-Wilk test. The mean and standard deviation were used to express descriptive statistics. To determine if there was a difference in the means of fracture resistance between the groups at p<0.05, analytical statistics that included an independent Student t-test were performed [13].

## Results

The present study contained 30 samples which were divided into two groups: primary and zigzag bevels. The data was analysed for normality by the Shapiro-Wilk test, and it was shown that the microleakage results of both groups were normally distributed. The distribution of the degree of microleakage for both groups is mentioned in Table 2.

**Table 2:** Distribution of the degree of microleakage amongst both the study groups

Degree of microleakage	Primary bevel		Zigzag bevel	
	N	%	N	%
0	0	0	10	66.7
1	8	53.3	5	33.3
2	7	46.7	0	0

In the primary bevel, 53.3% of the samples had a first degree of microleakage and 46.7% of the samples had a second degree of microleakage. In samples with zigzag bevels, 66.7% had no microleakage and 33.3% a first-degree microleakage. Comparing the mean difference of microleakage, the parametric independent Student t-test revealed that there was significantly less microleakage in the zigzag bevel than primary bevel at p<0.05, as shown in Table 3.

**Table 3:** Independent t-tests revealed significance in the distribution of the amount of microleakage between both groups

Bevel groups	Mean±SD	F	t	P-value
Primary bevel	1.46±0.51	1.544	6.178	0.000
Zigzag bevel	0.83±0.48			

Out of 30 samples, 10 samples (33.3%) did not show microleakage at all which was present in Group II (zigzag bevel) only. The first degree of microleakage was present in 13 samples (43.3%) which was distributed amongst both groups. The second degree of microleakage was present in seven samples (23.3%) which was distributed only in samples belonging to Group I (primary bevel).

**Figure 4:**
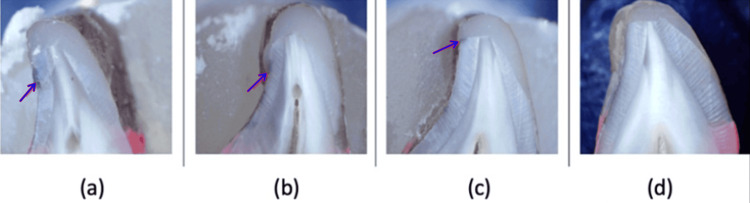
Microleakage as seen in a specimen from Group I (primary bevel)

Figure 4 represents a few samples depicting microleakage from Group I (primary bevel).

**Figure 5:**
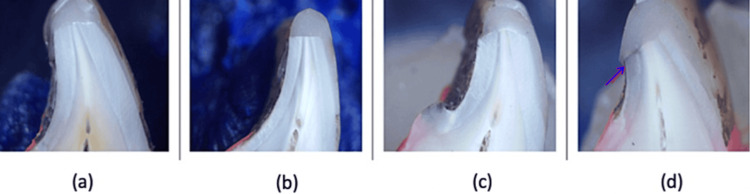
Microleakage as seen in a specimen from Group II (zigzag bevel)

Figure 5 represents a few samples depicting microleakage from Group II (zigzag bevel).

## Discussion

The definition of microleakage is "the clinically undetectable movement of bacteria, fluids, molecules or ions between tooth and the restorative or filling material" [14]. Numerous investigations highlight the fact that tooth-filling materials are dynamic microfissures rather than permanent, inert, and impenetrable walls that contain lively traffic of bacteria, ions, and chemicals. Although clinically invisible, this leakage represents a significant effect on the success rate of restorative therapy since it has numerous serious biological repercussions that can cause the pathology to be persistent and the procedure to fail [15].

In accordance with the explanation of microleakage provided above, microorganisms can enter the tooth/restoration interface through marginal spaces around a restoration. At a micron level, this is thought to be bacterial microleakage. Numerous studies have demonstrated that cariogenic bacteria are able to successfully spread along the tooth/restorative interface and have the potential ability to trigger an inflammatory adverse reaction from the pulpal tissue once they have gained access to this area. Recurrent caries is another effect of this phenomenon, which is one of the most common causes of bonding failures [16].

Although there are many causes of microleakage, one of the main causes of direct composite restoration failure is microleakage caused by polymerization shrinkage. The occlusal forces exert stress that could be detrimental to bond strength if voids formed due to polymerization shrinkage are present at the adhesive interface. An estimate of stresses reaching a range of 10 MPA has been made, enough to cause failure at the marginal region. Thus, a measure of microleakage can help quantify the effectiveness of the bonding of the restorative filling with the tooth material [17].

All dimensions of a cavity design like the quality of the substrate, available enamel for bonding, size, location of the margins, and mode of polymerization affect the shrinkage stress. The microleakage in composites can be found using a variety of techniques. Some of the most common techniques include dye penetration, dye extraction, the air pressure method, scanning electron microscopy (SEM), and micro-computed tomography [18].

The most popular method is to stain and identify microleakage/nanoleakage using coloured chemicals. The dye penetration approach involves staining contrasting dyes, such as immersion solution, to the interface to look for indications of staining. Notably, the most popular solutions are 50% silver nitrate, 2% methylene blue, and 0.5% basic fuchsin [19].

In comparison to other techniques, the dye penetration assay has some advantages. First off, there is no utilisation of radiation or reactive substances. Second, a range of dye solutions are available, which makes the process incredibly repeatable and practical. As dye penetration varies from area to area, evaluating just one portion of the tooth is not representative. Because the outcomes are more indicative of the leakage pattern, multiple-surface scoring methods are seen as being preferable to single-surface scoring methods.

As demonstrated in the present experiment, a stereomicroscope was utilised to assess the microleakage of the study samples. In the samples with primary bevel, all the samples exhibited microleakage of first and second degrees. In the samples with zigzag bevels, 66.7% did not have microleakage, and only 33.3% had first-degree microleakage. These results were in accordance with the results obtained by Coelho-De-Souza et al. in 2010 where they conducted experiments on bevel designs and microleakage in patients [20].

There are some limitations to the study. Firstly, the study design is a preclinical study or in vitro in nature. The study was done in a controlled environment, and it cannot be extrapolated to a clinical setting. Thermocycling was done to emulate the oral environment, but it can only replicate the clinical conditions to an extent. Another limitation is the limited sample size in this study. More studies should be done comparing the different types of bevel designs in vivo. More clinical research in this area needs to be done in order to make informed clinical decisions regarding the restorations of anterior fracture cases [21].

## Conclusions

Under the limitations of the present study, there was a statistically significant difference in microleakage observed, which favoured the samples that received the zigzag bevel as compared to the conventional primary bevel. Hence, to conclude, the zigzag bevel produced superior restorations with minimal marginal leakage than those restored with a primary bevel. Clinical trials should be performed to assess the performance of these bevel designs before definitive conclusions are formulated for clinical settings.
